# Oncogenic MicroRNAs Characterization in Clear Cell Renal Cell Carcinoma

**DOI:** 10.3390/ijms161226160

**Published:** 2015-12-08

**Authors:** Vincenzo Petrozza, Antonio Carbone, Teresa Bellissimo, Natale Porta, Giovanni Palleschi, Antonio Luigi Pastore, Angelina Di Carlo, Carlo Della Rocca, Francesco Fazi

**Affiliations:** 1 Pathology Unit, ICOT, Department of Medico-Surgical Sciences and Biotechnologies, Sapienza University of Rome, Latina 04100, Italy; vincenzo.petrozza@uniroma1.it (V.P.); natale.porta@alice.it (N.P.); carlo.dellarocca@uniroma1.it (C.D.R.); 2 Urology Unit, ICOT, Department of Medico Surgical Sciences and Biotechnologies, Sapienza University of Rome, Latina 04100, Italy; antonio.carbone@uniroma1.it (A.C.); giovanni.palleschi@uniroma1.it (G.P.); antonioluigi.pastore@uniroma1.it (A.L.P.); 3 Department of Anatomical, Histological, Forensic & Orthopaedic Sciences, Section of Histology & Medical Embryology, Sapienza University of Rome, Rome 00161, Italy; teresa.bellissimo@uniroma1.it; 4 Department of Medico-Surgical Sciences and Biotechnologies, Sapienza University of Rome, Latina 04100, Italy; angelina.dicarlo@uniroma1.it

**Keywords:** microRNAs, clear cell renal cell carcinoma, miR-21-5p and miR-210-3p

## Abstract

A key challenge for the improvement of clear cell renal cell carcinoma (ccRCC) management could derive from a deeper characterization of the biology of these neoplasms that could greatly improve the diagnosis, prognosis and treatment choice. The aim of this study was to identify specific miRNAs that are deregulated in tumor *vs.* normal kidney tissues and that could impact on the biology of ccRCC. To this end we selected four miRNAs (miR-21-5p, miR-210-3p, miR-185-5p and miR-221-3p) and their expression has been evaluated in a retrospective cohort of formalin-fixed paraffin-embedded (FFPE) tissues from 20 ccRCC patients who underwent surgical nephrectomy resection. miR-21-5p and miR-210-3p resulted the most significantly up-regulated miRNAs in this patient cohort, highlighting these onco-miRNAs as possible relevant players involved in ccRCC tumorigenesis. Thus, this study reports the identification of specific oncogenic miRNAs that are altered in ccRCC tissues and suggests that they might be useful biomarkers in ccRCC management.

## 1. Introduction

Renal cell carcinoma (RCC) is a common group of chemotherapy-resistant diseases and represents 2%–3% of adult malignancies, with the clear cell histotype (ccRCC) accounting for 80%–90% of all RCCs [1]. To date, the incidence of ccRCC has evidently increased and its mortality rate has reached 40%. Since ccRCC appear to be insensitive to chemotherapy and radiotherapy, the research of an effective post-operative adjuvant therapies is strongly needed [2].

A key challenge for the improvement of ccRCC management could derive from a deeper molecular characterization of these neoplasms that could greatly improve the diagnosis, prognosis and treatment choice [3]. In several tumors, miRNAs expression profile is emerging as a relevant marker for diagnosis, prognosis and treatment of cancer [4,5]. miRNAs are 22 nucleotides-long double strand small RNAs, typically excised from 60 to 110 nucleotide RNA precursor structures, which modulate gene expression generally at post-trascriptional level [6]. In fact, miRNAs show a developmental stage- and tissue-specific expression pattern and are present in complex regulatory circuits to regulate stem cells function, tissue differentiation and maintenance of cell identity during embryogenesis and adult life [7]. Notably, miRNA activity has also been correlated to the pathogenesis of cancer, since miRNAs have also been recently identified as a new class of genes with tumor-suppressor and oncogenic functions [8,9].

To date, a molecular characterization of ccRCC is under investigation and several high-throughput analyses have been recently performed in order to identify miRNAs putatively involved in ccRCC tumorigenesis and progression [10,11,12]. For example, miRNAs signatures are emerging as correlating with stage, grade and progression in ccRCC and, interestingly, a signature of 22 miRNAs that significantly correlated with patient survival was also recently described [13,14]. Moreover, specific miRNAs were identified to discern ccRCC from both papillary RCC (pRCC) and normal tissue [15]. Finally, a role of miRNAs in ccRCC metastatic progression is rapidly emerging as well [16,17,18,19].

Among the miRNAs showing a prognostic value in ccRCC, miR-21 and miR-210 demonstrated functional relevance for ccRCC tumorigenesis [20,21,22,23,24], as well as miR-185 and miR-221 [23,25].

By using a retrospective cohort of 20 formalin-fixed paraffin-embedded (FFPE) tissue samples, we evaluated the levels of specific miRNAs differentially expressed in ccRCC *vs.* matched normal tissues. We evidenced miR-21-5p and miR-210-3p as the most significantly up-regulated in this patient cohort, highlighting these onco-miRNAs as possible relevant players involved in ccRCC carcinogenesis.

## 2. Results

**Figure 1:**
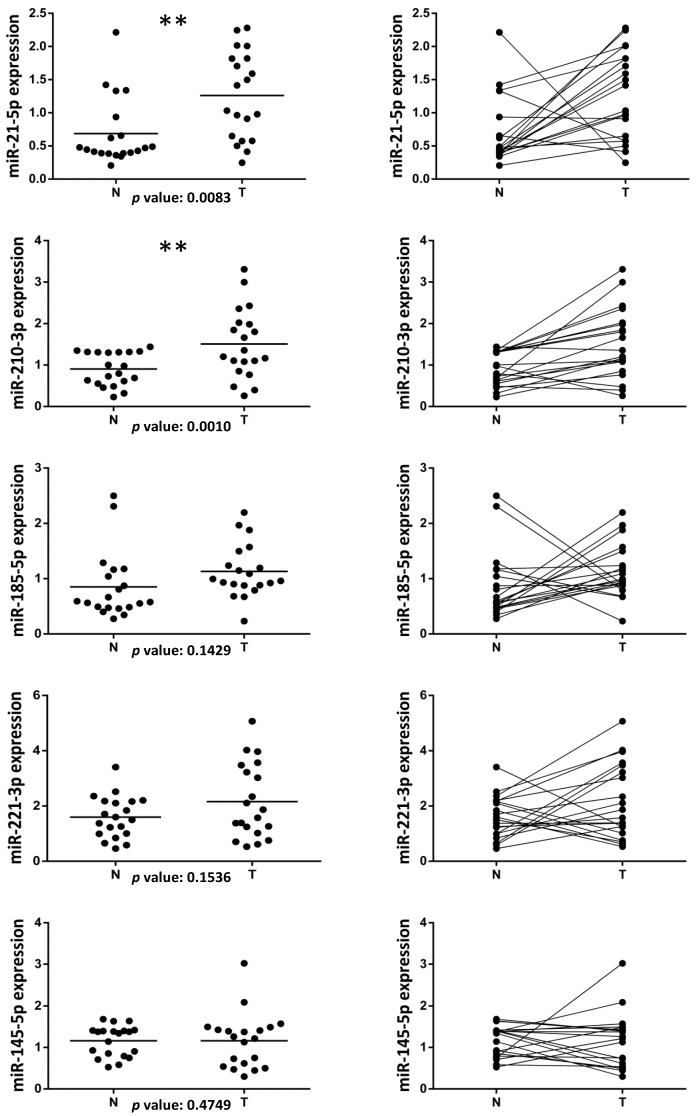
Evaluation of microRNAs levels in clear cell renal cell carcinoma (ccRCC) patients. Dot plots showing the expression of miR-21-5p, miR-210-3p, miR-185-5p and miR-221-3p and miR-145-5p in a retrospective cohort of formalin-fixed paraffin-embedded (FFPE) tissues from 20 ccRCC patients. A total of 20 matched ccRCC tumor (T) and adjacent normal tissue (N) samples were analyzed by RT-qPCR. The expression value of each miRNA was normalized over the average of RNU66, RNU19 and SCARNA17 expression. The *p* value was calculated by using a non-parametric Wilcoxon test with paired data and miRNAs whose differential expression was statistically significant (** *p* < 0.01) were indicated. Dot plots with the scatter of the individual data (**left** panel) or with connected lines between matched samples (**right** panel) are shown. Horizontal lines represent the median expression.

Among the miRNAs deregulated in several human cancers, we selected four miRNAs (miR-21-5p, miR-210-3p, miR-185-5p and miR-221-3p) to evaluate their expression in a retrospective cohort of formalin-fixed paraffin-embedded (FFPE) tissues obtained from 20 ccRCC patients undergoing surgical nephrectomy resection. The characteristics of ccRCC patients and tumor specimens are reported in the Patients and Methods section and summarized in Table 1. A total of 20 matched ccRCC and adjacent normal tissue samples were collected. Interestingly, miR-21-5p and miR-210-3p resulted significantly up-regulated in ccRCC *vs.* normal tissues, with a *p* value of 0.0083 and 0.0010, respectively (Figure 1). miR-185-5p and miR-221-3p, although did not show any statistically significant modulation between tumor and normal tissues, show a trend of expression similar to miR-21-5p and miR-210-3p (Figure 1). Moreover, we analyzed miR-145-5p expression that usually results particularly down-regulated in several tumor samples compared to normal tissues. We evidenced that miR-145-5p did not show any statistically significant modulation between tumor and normal tissues.

## 3. Discussion

In this study we observed that specific miRNAs, previously reported as up-regulated in ccRCC *vs.* autologous normal tissues, also show increased expression levels in our series of 20 FFPE tumor samples relatively to their matched normal counterparts. Specifically, among the up-regulated miRNAs, we confirmed increased levels of miR-21-5p, miR-210-3p, miR-185-5p and miR-221-3p. miR-21-5p and miR-210-3p resulted significantly up-regulated in this patient cohort highlighting these onco-miRNAs as relevant players involved in ccRCC tumorigenesis.

Interestingly, the increased expression of miR-21, miR-210, miR-185, miR-221 was previously reported in ccRCC patients and their contribution to ccRCC tumorigenesis is currently under investigation. miR-221 was significantly increased in ccRCC tissues and cell lines, while its knock-down inhibited cell proliferation, migration and invasion of renal cancer cells [25]. miR-210 was significantly overexpressed in ccRCC relatively to normal kidney and patients with high levels of miR-210 show a statistically higher incidence of disease recurrence [21]. Moreover, the down-regulation of miR-210 also reduced the migratory and invasive potential of metastatic RCC cells [22]. Using ccRCC and matched normal kidney samples, it was also evidenced that the increased levels of miR-185 and miR-21 in tumors correlate with the loss of function of specific tumor suppressors such as PTPN13, SLC12A1 and TCF21 [23]. Noteworthy is that miR-21 not only shows up-regulated expression in tumor tissues but also its serum levels resulted to be significantly correlated with the clinical staging of ccRCC patients [26].

In summary, this study confirms the deregulation of specific oncogenic miRNAs in ccRCC tissues and further supports the potential clinical usefulness of these miRNAs in ccRCC management.

## 4. Patients and Methods

### 4.1. Patients

This study was conducted on a retrospective cohort of ccRCC formalin-fixed paraffin-embedded (FFPE) tissue samples from 20 patients who underwent surgical resection between October 2011 and November 2013. For all the patients, FFPE-matched normal peritumoral kidney tissues were also considered. The patients were not treated with any neo-adjuvant therapy before surgery. Five patients were female (25%) and 15 patients were male (75%) with a mean age of 68.9 years old and a mean Body Mass Index (BMI) of 27.4 kg/m^2^. All the cases presented a clear cell histotype of RCC at the histological examination. The surgery procedures performed as treatments for these patients were: (i) open radical nephrectomy in 4 cases (20%); (ii) laparoscopic radical nephrectomy in 12 cases (60%); (iii) laparoscopic partial nephrectomy in 4 cases (20%). According to the tumor, node, and metastasis (TNM) classification, 10 patients have been identified as Stage I (50%), 5 patients have been identified as Stage II (25%), and 5 patients as Stage III (25%). Fuhrman’s grade has also been evaluated with 15% of cases belonging to the G1 grade (3 patients), 50% of cases belonging to G2 (10 patients) and 30% of cases to the G3 grade (6 patients). Only 1 patient actually showed a G2/3 grade. Complications have been classified as well and, according to the Clavien-Dindo classification, they have been identified only as grade I (75%) and grade II (25% of cases) [27]. Finally, main risk factors (such as hypertension, obesity and smoking habit) have been considered and findings showed that hypertension affected 9 patients (45%), obesity have been found in 5 (25%), and upon 20 patients, 7 were cigarette smokers; of the remaining 13 non-smokers, 4 have a history of tabagism (Table 1).

### 4.2. RNA Extraction and MicroRNA Expression Analysis

RNA from FFPE samples was extracted using the miRneasy^®^ FFPE kit (Qiagen, Chatsworth, CA, USA) following the manufacturer’s instructions. The concentration and purity of total RNA were assessed using a Nanodrop TM 1000 spectrophotometer (Nanodrop Technologies, Wilmington, DE, USA). A quantity of 150 ng of total RNA was reverse transcribed in 8 μL using miScript II RT kit (Qiagen, Chatsworth) and 1 μL of cDNA dilution (1:4) was used for quantitative real time PCR (RT-qPCR) experiments. PCR quantification analysis of the SCARNA17 and miRNAs miR-21-5p, miR-210-3p, miR-185-5p, miR-221-3p and miR-145-5p, was performed using the miScript SYBR Green PCR kit (Qiagen, Chatsworth) with the miScript Primer Assay Hs-SCARNA17 (#MS00014014), Hs-miR-21-5p (#MS00009079), Hs-miR-210-3p (#MS00003801), Hs-miR-185-5p (#MS00003647), Hs-miR-221-3p (#MS00003857), Hs-miR-145-5p (#MS00003528) (Qiagen, Chatsworth, CA, USA).

The expression analyses of RNU19 and RNU66 were performed by TaqMan MicroRNA RT assay and TaqMan MiRNA^®^ Assays (RNU19 #001003 and RNU66 #001002) (Applied Biosystems, Foster City, CA, USA) according to the manufacturer’s protocol.

All reactions were performed in duplicate. Data were analyzed by quantification relatively to a standard curve. The standard curve was prepared with serial dilutions of a reference cDNA obtained from RNA extracted from a tumor sample. z-scores were calculated for all expression values to standardize the data. Subsequently, z-score values of RNU66, RNU19 and SCARNA17 were averaged and used to normalize the expression values of each miRNA. The *p* value was calculated by using a non-parametric Wilcoxon test with paired data and miRNAs whose differential expression was statistically significant (** *p* < 0.01) was indicated.

**Table 1:** Clinical characteristics of patients with clear cell renal cell carcinoma (ccRCC).

Gender/Age	BMI	Clavien	Hyperten.	Smok. Habit	Tumor Size	Nodal Status	Metast.	TNM Stage	Histology	Tumour Cells (%)	Grade	Surgery
M/65	33.2	II	No	No	T2a	Nx	Mx	II	ccRCC	87	G2	O. Rad. Neph
F/65	22.3	I	No	Yes	T1a	Nx	Mx	I	ccRCC	89	G2/3	L. Rad. Neph
M/61	25.9	I	No	Former-15 yrs	T2	N0	Mx	II	ccRCC	90	G3	O. Rad. Neph
M/68	29.9	I	Yes	Former-30 yrs	T3a	Nx	Mx	III	ccRCC	84	G3	O. Rad. Neph
M/82	21.9	I	No	No	T3a	Nx	Mx	III	ccRCC	86	G3	L. Rad. Neph
M/84	24.6	I	No	No	T2a	Nx	Mx	II	ccRCC	87	G2	O. Rad. Neph
M/59	28.4	I	Yes	Yes	T1a	Nx	Mx	I	ccRCC	88	G2	L. Part. Neph
M/83	30.7	II	No	No	T1a	N0	Mx	I	ccRCC	90	G2	L. Rad. Neph
M/69	28.3	I	Yes	No	T2b	Nx	Mx	II	ccRCC	90	G2	L. Rad. Neph
M/55	23.14	I	Yes	No	T2b	Nx	Mx	II	ccRCC	90	G2	L. Rad. Neph
F/72	25.59	I	No	Yes	T1b	Nx	Mx	I	ccRCC	88	G2	L. Rad. Neph
M/65	40.3	II	No	Yes	T1a	Nx	Mx	I	ccRCC	87	G1	L. Part. Neph
F/59	31.4	I	Yes	Former-15 yrs	T1a	Nx	Mx	I	ccRCC	86	G1	L. Part. Neph
M/63	26.5	II	No	Yes	T1b	N0	Mx	I	ccRCC	85	G2	L. Rad. Neph
F/87	22.3	I	Yes	No	T3a	N0	Mx	III	ccRCC	88	G3	L. Rad. Neph
F/56	30.2	II	Yes	Yes	T1a	Nx	Mx	I	ccRCC	89	G2	L. Rad. Neph
M/64	24.3	I	No	No	T3a	Nx	Mx	III	ccRCC	88	G3	L. Rad. Neph
M/82	26.65	I	Yes	No	T3a	Nx	Mx	III	ccRCC	90	G3	L. Rad. Neph
M/77	29.46	I	Yes	Yes	T1a	Nx	Mx	I	ccRCC	88	G1	L. Part. Neph
M/66	24.71	I	No	Former-25 yrs	T1a	Nx	Mx	I	ccRCC	89	G2	L. Rad. Neph

M: Male; F: Female; BMI: Body Mass Index; Clavien: Clavien-Dindo Classification; Hyperten: Hypertension; Smok. Habit: Smoking Habit; Metast: Metastasis; TNM: Tumor, Node, and Metastasis Classification; yrs: years; T: Tumor Size; Nx: Regional Lymph Nodes cannot be assessed; N0: No Regional Lymph Node metastasis; Mx: Distant Metastasis cannot be evaluated; G: Grade; O. Rad. Neph: Open Radical Nephrectomy; L. Rad. Neph: Laparoscopic Radical Nephrectomy; L. Part. Neph: Laparoscopic Partial Nephrectomy.
